# Perspectives on plant virus diseases in a climate change scenario of elevated temperatures

**DOI:** 10.1007/s44154-022-00058-x

**Published:** 2022-09-06

**Authors:** Wei-An Tsai, Christopher A. Brosnan, Neena Mitter, Ralf G. Dietzgen

**Affiliations:** Centre for Horticultural Science, Queensland Alliance for Agriculture and Food Innovation, The University of Queensland, St. Lucia, QLD 4072 Australia

**Keywords:** Elevated temperatures, Virus epidemics, Plant virus control, RNA-based approaches, Nanoparticles

## Abstract

Global food production is at risk from many abiotic and biotic stresses and can be affected by multiple stresses simultaneously. Virus diseases damage cultivated plants and decrease the marketable quality of produce. Importantly, the progression of virus diseases is strongly affected by changing climate conditions. Among climate-changing variables, temperature increase is viewed as an important factor that affects virus epidemics, which may in turn require more efficient disease management. In this review, we discuss the effect of elevated temperature on virus epidemics at both macro- and micro-climatic levels. This includes the temperature effects on virus spread both within and between host plants. Furthermore, we focus on the involvement of molecular mechanisms associated with temperature effects on plant defence to viruses in both susceptible and resistant plants. Considering various mechanisms proposed in different pathosystems, we also offer a view of the possible opportunities provided by RNA -based technologies for virus control at elevated temperatures. Recently, the potential of these technologies for topical field applications has been strengthened through a combination of genetically modified (GM)-free delivery nanoplatforms. This approach represents a promising and important climate-resilient substitute to conventional strategies for managing plant virus diseases under global warming scenarios. In this context, we discuss the knowledge gaps in the research of temperature effects on plant-virus interactions and limitations of RNA-based emerging technologies, which should be addressed in future studies.

## Introduction

Global warming has been predicted to continually worsen this century due to increasing greenhouse gases in the atmosphere (Velasquez et al. 2018). According to a report from the Intergovernmental Panel on Climate Change (IPCC), the global mean surface temperature is likely to increase by 0.3–4.8 °C by the end of the twenty-first century (2081–2100) (IPCC 2014). Moreover, this increase in temperature is predicted to be a vital climate stressor that will affect food security globally (Mbow et al. 2019). Depending on the temperature optimum and geographical distribution of a crop species, elevated temperatures can have negative or positive effects on crop production. In a recent study on food security, elevated temperatures during crop growing seasons were found to adversely affect crop production in many regions that cultivate the top ten globally important crops for human sustenance (Ray et al. 2019). Elevated temperatures can affect crop yields in direct and indirect ways (DeLucia et al. 2012; Grulke 2011; Hatfield and Prueger 2015; Jeger et al. 2018; Mittler et al. 2012; Sun et al. 2017; Wahid et al. 2007). Directly, elevated temperatures cause production losses by interfering with plant physiological processes, plant development and reproduction (Hatfield and Prueger 2015; Mittler et al. 2012; Wahid et al. 2007). Indirectly, elevated temperatures affect crop yield through changes in plant disease progression or insect pest biology (DeLucia et al. 2012; Grulke 2011; Jeger et al. 2018; Sun et al. 2017).

Viruses account for almost 50% of emerging plant diseases worldwide (Jones and Naidu 2019). They decrease marketable quality of cultivated crops and result in yield losses at an estimated cost of more than $30 billion annually (Jones and Naidu 2019). As global temperatures continue to rise, plant virus diseases are likely to become more difficult to control (Jones 2009; Jones 2014; Jones 2016; Jones and Naidu 2019). For example, at elevated temperatures, some tomato spotted wilt virus (TSWV) isolates were shown to overcome the *Tsw* natural host resistance in capsicum plants (de Ronde et al. 2019). In addition, elevated temperatures may cause geographic shifts in the distribution of arthropod vectors that transmit plant viruses to cultivated plants and reservoir hosts such as weeds, thereby speeding up development of viral epidemics and thwarting established virus disease management measures (Jones 2016; Jones and Naidu 2019). Therefore, the predicted impact of elevated temperatures on virus epidemics needs to be comprehensively investigated to effectively manage plant virus diseases under a global warming scenario.

Considering a hierarchical system leading to virus epidemics, it is critical to understand virus infections at all nested levels from micro- to macro-environment, including cellular and molecular levels, individual plant level, plant population level and ecosystem level (Garrett et al. 2006; Jeger 2020). At the ecosystem level, changing epidemiological factors, such as arthropod vector populations, can make virus diseases a vital challenge to secure food production. Furthermore, at the molecular level, the diverse genetic background of host plants and evolving viral pathogens can make the outcomes of plant-virus interactions difficult to predict. Therefore, in this review, we provide a brief overview of how elevated temperatures may affect virus epidemics at micro- and macro-climate levels. Moreover, we focus on the underlying mechanisms involved in the effects of elevated temperatures on plant-virus interactions at the cellular and molecular levels. By synthesizing current knowledge of molecular mechanisms involved in viral pathosystems at elevated temperatures, we will elucidate potential approaches for sustainable plant virus disease management. Such understanding will be crucial for future research in developing disease control strategies that are resilient to climate variability.

## Effect of elevated temperatures on plant virus epidemics at micro- to macro-climate levels

The emergence and evolution of viruses is tightly controlled by interactions with their hosts and vectors under diverse environmental conditions from the micro level to global scale (Jones 2009; Lefeuvre et al. 2019). In plant pathology, the well-known “disease triangle” concept was proposed to analyse the development of plant diseases by incorporating variations in hosts, pathogens, and environments (Stevens 1960; Velasquez et al. 2018). Considering that in nature, 80% of plant viruses are transmitted by arthropod vectors (Hohn 2007), these vectors are likely critical factors in disease outbreaks. This led to a new conceptual model of tripartite plant-virus-vector interactions with environmental conditions as the underlying, connecting factor (Jones 2014; Jones and Naidu 2019).

Based on plant virus epidemiology studies, the magnitude of virus disease outbreaks depends on the number of primary infections, the spread of infections within hosts, and the transmission efficiency of the virus to new hosts resulting in secondary infections (Canto et al. 2009). Moreover, environmental variables are vital factors that can affect plants, viruses, virus vectors, and their interactions, which in turn determine progression of virus epidemics (Canto et al. 2009; Jones and Naidu 2019). Rising temperature, one of the environmental variables, is expected to significantly shape virus epidemics and to affect the severity or incidence of virus diseases (Chappell et al. 2013; Hunjan and Lore 2020; Jeger et al. 2018; Nancarrow et al. 2014; Reynaud et al. 2009). For example, the incidence of TSWV in summer months was associated with the average temperature in winter of the previous year. Since higher temperatures during winter enhanced the persistence of weeds that act as virus reservoirs and insect shelters, larger thrips populations were established earlier, which lead to a higher TSWV incidence (Chappell et al. 2013; Jeger et al. 2018).

### Effect of elevated temperatures on the fitness of viruses in their plant hosts

In natural ecosystems, many steps are crucial for viruses to maintain their fitness and complete their life cycle (Canto et al. 2009). Viral fitness is a measure of reproductive success of a certain viral genotype in a given environment (Elena and Lalić 2013). Within an individual host, interaction between plants and viruses is an important factor affecting the extent of viral fitness (Elena and Lalić 2013). Since viruses are intracellular obligate parasites, the effectiveness of viruses to hijack cellular components for their own benefit, reprogram host gene expression, and counteract cellular defences may contribute to a higher viral fitness (Elena and Lalić 2013).

During virus infection, unpacking and packaging of virus genome, replication, and cell-to-cell and systemic movement, are key steps in the infection cycle in individual plants (Elena and Lalić 2013). These steps can be influenced by cellular components and the interactions between viral and host components (Elena and Lalić 2013), which may lead to different viral fitness. Changes in temperature have been found to affect some of those steps, such as cell-to-cell and systemic movement and replication, which in turn impact within-host virus spread (Amari et al. 2021; Canto et al. 2009; Jones 2016). In general, the rates of virus replication and systemic movement increase as temperature increases. However, this initial upward trend of virus propagation likely attenuates when temperatures increase beyond the optimum of a particular virus (Table 1) (Amari et al. 2021). For example, the multiplication rate of tobacco mosaic virus (TMV) in *Nicotiana tabacum* leaf disks increased as the temperature rose from 20 °C to 32 °C, but was inhibited above 32 °C (Lebeurier and Hirth 1966). Similarly, the rate of systemic infection of potato virus Y (PVY) in *Solanum tuberosum* L. cv. Chu-Baek increased with increasing temperature from 20 °C to 28 °C (Choi et al. 2017). However, systemic infection of PVY ceased when the thermal threshold of 35 °C was surpassed (Choi et al. 2017). In *Brassica campestris*, the rate of systemic infection by turnip mosaic virus (TuMV) was shown to increase in a linear fashion as temperature increased from 13 °C to 23 °C (Chung et al. 2015). Moreover, TuMV-infected plants developed symptoms earlier and accumulated more viral coat protein (CP) at higher temperatures of 23 °C to 28 °C. On the other hand, delayed symptoms and lower accumulation of CP in TuMV-infected plants were observed as the temperature reached 33 °C. The systemic infection rate (the reciprocal of the systemic infection time) of TuMV at 33 °C was also lower than the rate predicted from the linear model (Chung et al. 2015). Besides temperature effects on the enhancement of TuMV replication, cell-to-cell and systemic movement of TuMV in *B. napus* was also found to be increased at 28 °C compared to 24 °C (Amari et al. 2021). A modified activity or turnover of viral movement proteins (MP) at elevated temperatures was proposed as the mechanism of this temperature-mediated enhancement of within-host virus spread (Amari et al. 2021; Boyko et al. 2000). MP are encoded in most plant virus genomes and function in virus intercellular movement (Kumar and Dasgupta 2021). During TMV infection, a viral ribonucleoprotein (vRNP) complex, containing viral RNA and MP, is formed at the peripheral endoplasmic reticulum (ER) and incorporates the replicase to form a virus replication complex (VRC) (Kumar and Dasgupta 2021). Subsequently, with the help of different host factors, this VRC may move along the ER-actin networks with support of myosin motor proteins to the plasmodesmata (PD). When VRC reaches PD, the MP was speculated to interact with PD-associated host factors to increase size exclusion limit (SEL) of PD and facilitate TMV intercellular spread (Kumar and Dasgupta 2021). At late stages of TMV infection, a microtubule-associated factor, MPB2C, is expressed to enhance accumulation of MP on microtubules (Curin et al. 2007; Kumar and Dasgupta 2021). Then the ATP-driven CDC48 is activated by the accumulation of MP, which leads to the CDC48-mediated delivery of MP to cytoplasm (Pitzalis and Heinlein 2017). This transfer of MP from ER to cytoplasm is critical for MP degradation by 26S proteosome, which may be important for relieving ER stress and returning ER to its pre-infection morphology (Pitzalis and Heinlein 2017). Elevated temperature could potentially facilitate TMV spread by increasing myosin motor activity (Amari et al. 2021). Furthermore, an increased association of MP with microtubules has been shown to be correlated with the enhanced cell-to-cell movement of TMV at elevated temperature (Boyko et al. 2000). Since no MP degradation was reported, changes of MP activity and intracellular distribution at elevated temperatures are likely to contribute to the observed enhanced virus movement (Amari et al. 2021; Boyko et al. 2000).

**Table 1:** Effects of elevated temperatures on plant susceptibility to viruses

Viruses	Temp	Temp optimum (plant vegetative growth/virus replication or symptom)	Phenotype	Accumulation of virus or virus-derived nucleic acids at HT compared to NT or LT	Proposed mechanisms / components	References
CymRSV	27 °C	23.5 °C (Desaint et al. 2021)/n.d.	Symptom attenuation at elevated temp in wt *N. benthamiana*	Viral gRNA, sgRNA and vsiRNA levels in protoplasts: 27 °C > 15 °C	RNA silencing	(Szittya et al. 2003)
TCV, PVX, TMV	27 °C	23.5 °C (Desaint et al. 2021)/20–24 °C (PVX) (Close 1964)	Symptom attenuation at elevated temp in wt *N. benthamiana*	Viral gRNA levels in systemic leaves at 14 dpi: 27 °C < 21 °C	RNA silencing /NbRDR6	(Qu et al. 2005)
TMV (common strain)	35 °C	23.5 °C (Desaint et al. 2021) /24 °C (Lebeurier and Hirth 1966)	Increased multiplication of TMV as temp rising from 20 °C to 32 °C Blocked TMV replication in *N. tabacum* leaf disks at 35 °C	No viral RNA detected in tobacco leaf disks treated at 35 °C	Blocking of viral replication	(Lebeurier and Hirth 1966)
TCV	26 °C	22–23 °C (Desaint et al. 2021)/n.d.	TCV-infected dcl2 or dcl2/dcl3, ago2–1 or ago1–27/ ago2–1, and hen1 *A. thaliana* mutants did not survive at 26 °C, while TCV-infected wt *A. thaliana* did	Viral gRNA and vsiRNA levels in systemic leaves at 7 dpi: 26 °C > 18 °C)	RNA silencing / AtDCL2, AtAGO2, and AtHEN1	(Zhang et al. 2012)
ToRSV	27 °C	23.5 °C (Desaint et al. 2021)/n.d.	Symptom recovery at elevated temp in wt *N. benthamiana*	• Viral RNA2 and vsiRNA levels in inoculated and systemic leaves at early stages of infection (3,4,5 dpi): 27 °C > 21 °C • Viral RNAs levels at later stages of infection (6 dpi): 27 °C = 21 °C	RNA silencing (translation inhibition) / AGO1	(Ghoshal and Sanfacon 2014)
TRSV	26–29 °C or 33 °C	23.5 °C (Desaint et al. 2021)/n.d.	Symptom attenuation at 33 °C in wt *N. tabacum* cv Xanthi Blocked systemic infection of TRSV in wt *N. tabacum* at temp above 26 °C	• Viral RNA levels in uppermost leaves at 10 and 35 dpi: 33 °C < 25 and 18 °C • Viral RNA levels in systemic leaves at 35 dpi: VSR (HCPro and AC2) expressing transgenic plants>wt plants at 29 °C	RNA silencing	(Siddiqui et al. 2008)
CMV	28 °C	23.5 °C (Desaint et al. 2021) /n.d.	*N. tabacum* displayed severe symptoms at early stage (6 dpi) at HT of 28 °C than at NT of 24 °C and LT of 18 °C Recovery phenotype (dark green island) displayed earlier (12dpi) at HT of 28 °C	• CMV-CP RNA levels in inoculated leaves at 6 dpi: 28 °C > 24 and 18 °C • CMV-CP RNA levels in systemic leaves at 28 dpi: 28 °C < 24 and 18 °C • VsiRNA levels from 6 dpi to 12 dpi: 28 °C > 24 and 18 °C	RNA silencing	(Zhao et al. 2016)
CPsV	32/26 °C, day/night	25–30 °C (Abobatta 2019)/n.d.	Symptom attenuation at elevated temp in *Citrus sinensis*	• Viral RNA levels: 32/26 °C < 26/18 °C • VsiRNA levels: 32/26 °C > 26/18 °C	RNA silencing	(Velázquez et al. 2010)
Recovery type: ACMV, SLCMV Non-recovery type: EACMCV, EACMV	30 °C	29.9 ± 0.6 °C (Parent and Tardieu 2012)/n.d.	Symptom recovery at elevated temp in cassava infected with non-recovery type cassava mosaic geminiviruses	• Viral DNA levels of ACMV, SLCMV, EACMCV, and EACMV in symptomatic young leaves at 2wpi, 3wpi, 4wpi and 5wpi: 30 °C < 25 and 25–30 °C) • VsiRNA levels of ACMV, SLCMV, EACMCV, and EACMV in symptomatic young leaves at 2wpi, 3wpi, 4wpi and 5wpi: 30 °C > 25 and 25–30 °C	RNA silencing	(Chellappan et al. 2005)
PVY	28 °C	15–25 °C (Rykaczewska 2015)/28 °C (Close 1964)	Heat-enhanced symptoms in susceptible *Solanum tuberosum* (cv. Chicago)	• Higher levels of viral RNA in systemic leaves of PVY-susceptible potato than in PVY-resistant potato • Viral RNA levels in systemic leaves of PVY-susceptible potato: 28 °C > 22 °C	Suppression of SA-induced defence genes	(Makarova et al. 2018)
	35 °C	15–25 °C (Rykaczewska 2015)/28 °C (Close 1964)	Increased systemic infection of PVY in *S. tuberosum* (cv. Chu-Baek) as temp rising from 20 °C to 28 °C Heat-blocked systemic infection of PVY in *S. tuberosum* (cv. Chu-Baek) at 35 °C	PVY undetectable by ELISA in systemic leaves at thermal threshold of 35 °C	Unknown mechanism	(Choi et al. 2017)
TYLCV	40–45 °C /20–25 °C, day/night	18–25 °C (Desaint et al. 2021)/n.d.	Heat-enhanced symptoms in susceptible *S. lycopersicum* (Line 967)	Viral DNA levels in systemic leaves of Line 967: 40–45 °C /20–25 °C > 22–25 °C /18–20 °C	Less HSPs, HSFs and APXs induced in susceptible Line than resistant Line at HT	(Anfoka et al. 2016)
TuMV	32 °C /28 °C, day/night	22–23 °C (Desaint et al. 2021)/n.d.	Heat-enhanced symptoms in *A. thaliana*	n.d.	Different expression pattern of R genes and basal defence genes	(Prasch and Sonnewald 2013)
	33 °C	20–30 °C (Singh et al. 1982)/23–28 °C for symptom expression (Chung et al. 2015)	Enhanced CP RNA accumulation of TuMV in *Brassica campestris* as temp rising from 23 °C to 28 °C Decreased systemic infection of TuMV in *B. campestris* at 33 °C	Accumulation of CP RNA: 33 °C < 28 °C and 23 °C	Unknown mechanism	(Chung et al. 2015)
	28 °C	23.1 ± 0.7 °C (Parent and Tardieu 2012)/n.d.	Increased cell-to-cell and systemic movement of TuMV in *B. napus* at 28 °C		Increased cell-to-cell and systemic movement	(Amari et al. 2021)
GBNV	30 and 25 °C	33.1 ± 0.9 °C (Parent and Tardieu 2012)/n.d.	Heat-enhanced symptoms in *V. unguiculata* L.	Viral RNA levels in inoculated leaf: 30 and 25 °C > 15 and 20 °C	Higher accumulation of H_2_O_2_ and cell death at HT (30 and 25 °C)	(Singh et al. 2018)
TSWV	29/24 °C, day/ night	23.5 °C (Desaint et al. 2021)/n.d.	Heat-enhanced symptom severity in *N. tabacum*	Days required for detection of TSWV in different organs of tobacco: 29/24 °C < 23/18 °C	Heat-enhanced TSWV translocation only in tobacco	(Llamas-Llamas et al. 1998)
	35 °C	n.d./n.d.	Heat-masked symptoms in *Ficus* spp.	n.d.	Unknown mechanism	(Lavina and Battle 1993)
INSV	33 °C	20–25 °C (Desaint et al. 2021)/n.d.	Heat-blocked systemic infection in *C. annuum*, *C. chinense* PI152225, and *C. chinense* PI159236	INSV undetectable by ELISA in systemic leaves of capsicum plants at 33 °C	Unknown mechanism	(Roggero et al. 1999)
CaCV	35 °C	20–25 °C (Desaint et al. 2021)/n.d.	Symptom recovery at elevated temp in *C. annuum*	• Viral RNA in systemic leaves at 18 dpi: 35 °C < 25 °C • vsiRNA in systemic leaves at 10 dpi: 35 °C > 25 °C	RNA silencing	(Tsai et al. 2022)

*Temp* Temperature, *HT* Higher temperature, *NT* Normal temperature, *LT* Lower temperature, *wt* Wild type, *N. benthamiana Nicotiana benthamiana*, *N. tabacum Nicotiana tabacum*, *n.d.* Not determined, *CymRSV* Cymbidium ringspot virus, *TCV* Turnip crinkle virus, *PVX* Potato virus X, *dpi* Days post inoculation, *wpi* Weeks post inoculation, *TMV* Tobacco mosaic virus, *ToRSV* Tomato ringspot virus, *TRSV* Tobacco ringspot virus, *CMV* Cucumber mosaic virus, *CP* Coat protein, *CPsV* Citrus psorosis virus, *ACMV* African cassava mosaic virus, *SLCMV* Sri Lankan cassava mosaic virus, *EACMCV* East African cassava mosaic Cameroon virus, *EACMV* East African cassava mosaic virus, *PVY* Potato virus Y, *TYLCV* Tomato yellow leaf curl virus, *TuMV* Turnip mosaic virus, *GBNV* Groundnut bud necrosis virus, *TSWV* Tomato spotted wilt virus, *INSV* Impatiens necrotic spot virus, *CaCV* Capsicum chlorosis virus

Since plants as virus hosts provide the environment for viruses, plant fitness is equally important for the outcome of plant-virus interactions at elevated temperature. Generally, many of the crops that are classified in the group of mesophilic higher plants grow best at temperatures ranging from 10 to 30 °C (Luo 2011; Nievola et al. 2017). When ambient temperatures exceed the optimum growth range of a particular plant species, impacts on plant metabolism and functions may occur (Nievola et al. 2017). Elevated temperatures are therefore considered as stresses, which can affect plant physiological and biochemical processes, including, membrane fluidization, production of reactive oxygen species (ROS), accumulation of secondary metabolites and synthesis of osmolytes (Desaint et al. 2021; Nievola et al. 2017). Moreover, such changes may trigger other molecular mechanisms that affect plant fitness (Desaint et al. 2021; Venkatesh and Kang 2019). As mentioned above, viral fitness depends on the interactions between the viruses and the defence mechanisms of the plants (Elena and Lalić 2013). Elevated temperatures can enhance plant antiviral RNA silencing, which may lead to less severe symptoms on some virus-infected plants (Table 1) (Ghoshal and Sanfacon 2014; Qu et al. 2005; Szittya et al. 2003; Zhang et al. 2012). Evidence of the association between plant defence responses and viral fitness have been documented in TuMV-infected *Arabidopsis thaliana* and tobacco etch virus (TEV)-infected *N. tabacum* (Cervera et al. 2018). The plants in these studies were challenged with viruses with different extent of fitness. The transcriptomic profiles in these two studies indicated plant defence responses can be determined or reprogrammed by viral fitness (Cervera et al. 2018). In TEV-infected *N. tabacum*, the higher the viral fitness, the higher the activation of genes participating in hormone- and RNA silencing-mediated defence pathways (Cervera et al. 2018). Collectively, the results indicated that several defence responses, including RNA silencing, hormone, or effector-triggered immunity (ETI) are required for plants to mount strong defence to viruses (Cervera et al. 2018). In addition, evidence (which will be discussed in detail later) suggested that RNA silencing may be involved in abolished systemic infection of tobacco ringspot virus (TRSV) in *N. tabacum* at temperatures above 26 °C (Siddiqui et al. 2008). Based on the evidence mentioned above, the increase in host antiviral RNA silencing is likely to correlate with the negative effect of temperatures on viral fitness (i.e., virus replication) above a certain optimum. Elevated temperatures, on the other hand, can increase plant susceptibility to other viruses by negatively affecting plant resistance (R) gene-mediated or quantitative disease resistance (Table 2) (Marques de Carvalho et al. 2015; Romero et al. 2002; Wang et al. 2009; Whitham et al. 1996). This negative effect of elevated temperatures on plant resistance responses may be explained as less effective adaptation of plants than of pathogens to conditions above their optimum temperature range (Desaint et al. 2021). However, the underlying mechanisms involved in temperature-mediated resistance-breaking have rarely been studied in viral pathosystems and thus remain largely unknown. Potential options for addressing this will be discussed in the section starting on page 15.

At the macro-climate level, dynamics of virus populations is a critical factor affecting viral fitness (McLeish et al. 2020). An increase in temperatures may affect virus epidemics by changing geographical distribution of viruses (Jones 2014; Jones 2016). For example, potato leaf roll virus and potato yellow vein virus, which adapt well to higher temperatures, may be introduced to regions that were initially too cold for them (Jones 2014; Jones 2016). This geographical expansion of certain viruses may in turn change the viral population dynamics in that area since viruses can synergistically or antagonistically interact with each other during the process of infecting the same host (Alcaide et al. 2020; Martín and Elena 2009; Syller and Grupa 2016). Viruses introduced into new areas due to climate change may also allow them to interact with new hosts, which can lead to new epidemics (Jones and Coutts 2015). Elevated temperatures may provide optimal conditions for certain virus strains, which may in turn alter the spatial structure of the viral population by affecting virus evolution rates. An example of this is the emergence of naturally-selected resistance-breaking strains (de Ronde et al. 2019). The *Tsw* gene, a single dominant gene that confers resistance to TSWV in Capsicum spp., has been commercially used for several years (Moury et al. 1997; Moury et al. 2000). However, this resistance gene has been overcome by some TSWV isolates. Based on different degrees of virulence, TSWV isolates are classified into resistance-inducing (RI), temperature-dependent resistance-breaking (TempRB) or absolute resistance-breaking (AbsRB) (de Ronde et al. 2019). The RI, TempRB and AbsRB isolates were able to break *Tsw*-mediated resistance at temperatures above 32 °C, 28 °C and 23 °C, respectively (de Ronde et al. 2019). Interestingly, as all the TSWV isolates were collected from the Mediterranean region where high temperatures regularly occur, the authors suggested that viral adaptive evolution of resistance-breaking isolates is likely driven by elevated temperatures (de Ronde et al. 2019). They speculated that TSWV may enhance its virulence and fitness by firstly generating the TempRB isolate, which may subsequently evolve into an AbsRB isolate (de Ronde et al. 2019). Mixed virus infections may also affect viral population structure and evolution at elevated temperatures (Alcaide et al. 2021; Jones 2014; Jones 2016). For example, PVX alone failed to multiply and move out of the inoculated leaf of tobacco plants at 31 °C. However, a mixed PVX infection with other viruses, including TMV and CMV, allowed PVX to multiply and move systemically at that temperature (Close 1964). Very recently, the mixed infection of two strains of pepino mosaic virus (the European (EU) strain and Chilean (CH2) strain) was found to affect the genetic variability of both CH2 and EU populations at 30 °C, but not at 20 °C (Alcaide et al. 2021). Combined, all of the above provides clear evidence that elevated temperatures are an important climate factor that can shape viral evolution.

**Table 2:** Effects of elevated temperatures on plant resistance to viruses

Viruses	Temp	HT effects	Phenotypes shown at HT	Mechanisms involved in temperature effects	References
TMGMV-J	30 °C	HT break R gene (*L*^*1*^, *L*^*2*^) resistance in *C. annuum*	Virus moved systemically and caused necrosis on uninoculated leaves	unknown	(Sawada et al. 2004)
PaMMV-J	26 °C and 28 °C	HT break R gene (*L*^*1a*^) resistance in *C. annuum*	Vein necrosis and virus infection became systemic in L^1a^-homozygote	unknown	(Sawada et al. 2004)
Resistance-inducing isolate of TSWV	32 °C	HT break R gene (*Tsw*) resistance in *C. chinense*	Local HR was induced on inoculated leaves, but virus infection and TSWV typical symptoms became systemic	unknown	(de Ronde et al. 2019)
TSWV isolated from paprika	30 ± 2 °C	HT break R gene (*Tsw*) resistance in *C. chinense*	Local HR was induced on inoculated leaves, but necrotic spots developed on systemic leaves of 33% of PI15225 isolate / mottle symptoms developed on systemic leaves of 50% of S3669 isolate	unknown	(Chung et al. 2018)
TSWV	32 °C	HT break R gene (*Tsw*) resistance in *C. chinense*	Local HR was suppressed on inoculated leaves, and necrotic symptoms developed on systemic leaves of some plants	Heterozygous *Tsw* increased the appearance of systemic necrosis symptoms at 32 °C	(Moury et al. 1998)
PVY^NTN^	28 °C	HT break R gene (*Ny-DG*) resistance in *S. tuberosum* L.	Necrotic lesions developed on both inoculated and systemic leaves at 28 °C. Symptomless resistance to PVY^NTN^ was shown at 20 °C.	Increased expression of miR162, miR168a and miR482 and decreased expression of their target genes were shown in inoculated leaves at 28 °C	(Szajko et al. 2019)
PVX	30 °C	HT suppress R gene (*Rx*) -mediated HR in *N. benthamiana*	Local HR was suppressed on inoculated leaves	unknown	(Wang et al. 2009)
TMV	28 °C or 30 °C	HT suppress R gene (*N*) -mediated HR in *N. tabacum*	Local HR was suppressed on inoculated leaves	Decrease in N protein nuclear localization at 28 °C or 30 °C	(Wang et al. 2009; Zhu et al. 2010)
TriMV	24 °C	HT break R gene (*wsm3*) resistance in *T. aestivum*	Symptoms developed at 24 °C	unknown	(Liu et al. 2011)
WSMV	30 °C	HT break R gene (*wsm1*, *wsm2*) resistance in *T. aestivum*	Symptoms developed at 30 °C	unknown	(Liu et al. 2011)
TMV pathotype P_0_	34 °C	No suppression of *C. annuum* resistance to TMV- P_0_	Local HR was suppressed on inoculated leaves, but no symptom developed in systemic leaves	R-mediated resistance was suppressed by HT, but antiviral RNA silencing was enhanced to confer plant resistance	(Kim et al. 2021)

*HT* Higher temperature, *HR* Hypersensitive response, *Temp* Temperature, *n.d*. Not determined, *PVX* Potato virus X, *TMV* Tobacco mosaic virus, *PVY* Potato virus Y, *TSWV* Tomato spotted wilt virus, *TriMV* Triticum mosaic virus, *WSMV* Wheat streak mosaic virus, *TMGMV* Tobacco mild green mosaic virus, *PaMMV* Paprika mild mottle virus, *N. benthamiana Nicotiana benthamiana*, *N. tabacum Nicotiana tabacum*, *T. aestivum Triticum aestivum*, *C. annuum Capsicum annuum*, *C. chinense Capsicum chinense*, *S. tuberosum Solanum tuberosum*

### Effect of elevated temperatures on virus transmission

The efficiency of between-host transmission, including vertical and horizontal transmission, is another important component that determines viral fitness (Cobos et al. 2019; Elena and Lalić 2013). In vertical transmission, viruses are transmitted from the parent plant to the offspring through seeds (Cobos et al. 2019). The efficiency of the vertical or seed transmission has been reported to be affected by temperature in many cases (Sastry 2013). Moreover, this temperature effect varies depending on different plant-virus pathosystems (Sastry 2013). For example, barley stripe mosaic virus-infected barley plants grown at 20–24 °C showed greater seed transmission than those grown at higher or lower temperatures. On the other hand, 95% of the embryos were infected in southern bean mosaic virus-infected bean plants grown at 16–20 °C, while only 55% of embryos were infected in plants grown at 28–30 °C (Sastry 2013).

Horizontal transmission of viruses between host plants is mainly achieved by arthropod vectors (Jeger 2020). Depending on the required time of virus acquisition on infected plants, virus retention in vectors, and virus inoculation on recipient plants, vector transmission can be classified into four main modes: non-persistent, semi-persistent, circulative, non-propagative, and circulative, persistent-propagative (Dietzgen et al. 2016). The effects of these modes of transmission on insect-virus interactions and the outcomes of virus epidemics have been comprehensively reviewed (Dietzgen et al. 2016; Jeger 2020). Numerical methods were used to characterize virus epidemics in relation to these transmission modes based on the vector-incorporated susceptible-exposed-infectious-removed model (Madden et al. 2000). According to this deterministic simulation, significant differences in disease development among these modes of transmission were exhibited (Madden et al. 2000). Furthermore, vector performance (i.e., longevity, fecundity, and changes in population density) and behaviors (i.e., dispersal, landing and feeding preference) are considered important factors for progression of epidemics (Jeger 2020; Jeger et al. 2018; Madden et al. 2000; Sisterson 2009).

Virus transmission by vectors can also be influenced by numerous environmental factors (Jones 2016). Temperature is one of the predominant environmental variables that affects insect vector performance and behaviour, which may in turn influence viral transmission and spread (Jones 2016). Whiteflies and aphids, which are important plant virus vectors that impact global food security, react strongly to any minor changes of temperature. For example, *Bemisia tabaci*, benefits from warmer winter temperatures by increasing its distribution to those places that were formally too cold for its population to establish (Jones 2016). In temperate regions, increased temperatures favour aphid mobility by increasing the proportion of winged adults and their associated flight activity, which enables them to travel considerable distances (Hullé et al. 2010). Moreover, an increase in temperature of only 2 °C contributed to five additional generations of aphids per year due to their short generation time and high reproduction capacity (Hullé et al. 2010). These effects of elevated temperatures on either dispersal or reproduction of aphids would likely allow aphid-transmitted viruses to spread more widely in temperate climatic zones.

The risk of chili leaf curl virus (ChiLCV) disease in chili was correctly predicted based on simulation of temperature effects on *B. tabaci* abundance and transmission ability (Roy et al. 2021). The monthly temperatures correlated with the spatio-temporal distribution of vector abundance. This can in turn reflect leaf curl incidence caused by ChiLCV since the temperature thresholds (15–35 °C) that favour *B. tabaci* abundance and transmission ability of ChiLCV overlap (Roy et al. 2021). The predicted disease risk that reflects seasonal variation in disease incidence was validated by field surveys done prior to that study (Chaubey and Mishra 2017; Kumar et al. 2016). Overall, these findings demonstrate the importance of vector transmission in the progression of virus epidemics at elevated temperatures.

## Effect of elevated temperatures on plant-virus interactions at the molecular level in regard to host defence mechanisms

Elevated temperatures can affect plants, viruses, and their interactions, which may influence within-host virus dynamics (i.e., movement and accumulation) and in turn affect symptoms (Amari et al. 2021; Canto et al. 2009; Jones 2016). Notably, changes in virus accumulation in individual plants can influence virus transmission (Cobos et al. 2019; Jeger 2020). Extensive evidence has demonstrated a positive correlation between within-host viral accumulation and viral transmission rates (Froissart et al. 2010; Matsukura et al. 2013). Taken together, plant-virus interactions and their effects on within-host virus dynamics may be critical factors that affect plant virus disease epidemics. It is therefore important to understand the effect of elevated temperatures on plant-virus interactions to develop durable strategies for restraining virus dispersal within host plants or within fields under elevated temperature conditions.

### Effect of elevated temperatures on the interactions between susceptible plants and viruses

A well-known effect of elevated temperatures on the outcomes of the interactions between virus-susceptible plants and viruses was described as “temperature masking” a century ago (Johnson 1921). Even though this masked phenotype or symptom attenuation caused by elevated temperatures was reported in several viral pathosystems long ago (Hildebrand 1958; Lavina and Battle 1993), the studies on the molecular mechanisms involved in those cases are lacking. To date, some evidence suggested that antiviral RNA silencing is correlated with this attenuation of virus-induced symptoms. RNA silencing or RNA interference (RNAi) is a conserved antiviral defence response in plants (Ding and Voinnet 2007; Moon and Park 2016; Vaucheret 2006). The trigger of this defence response is the production of long double-stranded (ds) RNAs that are generated during virus replication, structured regions of RNA transcripts or bidirectional transcription of overlapping reading frames (Mlotshwa et al. 2008; Qin et al. 2017). Long dsRNAs are cleaved by dicer-like proteins (DCL) to yield 21–24 nt small interfering (si) RNA duplexes. In *A. thaliana*, DCL2 and DCL4 are the predominant DCLs for processing dsRNAs into 22 and 21 nt virus-derived siRNAs (vsiRNAs) in the defence against RNA viruses (Deleris et al. 2006; Qin et al. 2017). On the other hand, DCL3 is the main DCL that generates 24 nt vsiRNAs to silence DNA viruses (Mlotshwa et al. 2008). Once siRNA duplexes are formed, single-stranded (guide strand) RNA from the duplex is loaded onto Argonaute (AGO) proteins and incorporated into an RNA-induced silencing complex (RISC). Based on different AGO proteins that are incorporated into RISC, post transcriptional gene silencing (PTGS) or transcriptional gene silencing (TGS) are propagated through the plant to silence viruses by slowing virus accumulation and systemic movement (Mallory and Vaucheret 2010; Muhammad et al. 2019).

In many cases, antiviral RNA silencing was enhanced at elevated temperature, which in turn led to symptom attenuation in virus-susceptible plants (Table 1). For example, symptoms were attenuated in *N. benthamiana* infected with cymbidum ringspot virus (CymRSV) at higher temperature of 27 °C but not below 24 °C (Szittya et al. 2003). In this study, attenuated symptoms on CymRSV-infected plants were accompanied by reduced virus accumulation and increased vsiRNAs at the elevated temperature (Szittya et al. 2003). Furthermore, Cym19stop mutant, which encodes a non-functional P19 viral silencing suppressor (VSR) (Silhavy et al. 2002), was found to cause less severe symptoms compared to those of wildtype CymRSV-infected plants at 21 °C and 24 °C. In addition, the spread of Cym19stop was blocked at 21 °C and 24 °C while CymRSV was able to spread by suppressing RNAi (Szittya et al. 2003). In addition, symptom attenuation was also observed in TRSV-inoculated *N. tabacum* at 33 °C. The systemic infection of TRSV, which was abolished in wt *N. tabacum*, was established in VSR (HC-Pro and AC2) transgenic *N. tabacum* at 26–29 °C (Siddiqui et al. 2008). This suggested a possible involvement of increased RNAi in symptom attenuation and a blockage of systemic virus infection at elevated temperatures (Siddiqui et al. 2008). Subsequently, several critical components, including RNA-dependent RNA polymerase 6 (RDR6), DCL2, AGO2 and Hua Enhancer 1 (HEN1), were all shown to contribute to the increased antiviral RNAi at elevated temperatures in different pathosystems (Qu et al. 2005; Zhang et al. 2012).

Symptom recovery has been widely used to describe the emergence of young asymptomatic leaves following an initial systemic symptomatic infection (Ghoshal and Sanfacon 2015). This change in symptom phenotype can be induced by elevated temperature. The first report of this phenomenon was published in 1928, in which virus titre appeared to be reduced in recovered leaves of tobacco rattle virus (TRV)-infected *N. tabacum* (Cadman and Harrison 1959; Ratcliff et al. 1999a; Wingard 1928). In subsequent studies, the recovered plants were shown to be resistant to secondary infection by related viruses in a sequence-specific manner, suggesting that RNA silencing is involved in symptom recovery (Paudel et al. 2018; Ratcliff et al. 1997; Ratcliff et al. 1999b; Santovito et al. 2014). Moreover, an association between RNA slicing and plant recovery was reported in *N. benthamiana* infected with p19-deficient tomato bushy stunt virus (TBSV) (Omarov et al. 2007). Viral RNA clearance followed by recovery was shown in the infection of TBSV p19 mutant. In this study, vsiRNA-containing protein complexes extracted from recovered leaves were shown to have sequence-specific RNase activity (Omarov et al. 2007). This implies that RISC-directed RNA slicing may be involved in symptom recovery. However, a concomitant viral nucleic acid reduction is not strictly required (Ghoshal and Sanfacon 2014; Ghoshal and Sanfacon 2015; Kørner et al. 2018). An example was shown in *A. thaliana* plants infected with the tobamovirus oil-seed rape mosaic virus (ORMV), which underwent natural recovery 23–25 days post-infection (dpi). Results showed that the amount of full-length viral RNA was similar in all infected tissues, including recovered leaves. However, when green fluorescent protein (GFP)-silenced transgenic plants were infected with ORMV that encodes the strong VSR p125, a non-fluorescent phenotype was established in recovered leaves and a fluorescent phenotype persisted in symptomatic mature leaves. These results suggest that recovery from symptoms was concomitant with reduction of VSR activity, but not with viral RNA clearance (Kørner et al. 2018). A collection of *A. thaliana* RNAi mutants were inoculated with ORMV to observe disease symptom development (Kørner et al. 2018). The results showed that mutations in RNAi pathway genes, including *ago1*, *hen1*, *dcl4*, *rdr6*, *suppressor of gene silencing 3* (*sgs3*), *rdr2*, and *nuclear RNA polymerase D* (*nrpd*) (Pol IV subunit) led to non-recovered or weakly recovered plants. On the contrary, enhanced recovery was observed in *dcl3* mutant plants. In addition, this enhanced recovery was impaired in *dcl3- dcl4* double mutants. This suggests that the recovery is associated with DCL4-dependent PTGS instead of DCL3-dependent TGS. Interestingly, even though recovery symptoms were independent of TGS pathway, the involvement of the other two TGS components, RDR2 and PolIV is required. Given the important function of RDR2 and PolIV in maintenance and spread of silencing (Dunoyer et al. 2007), Kørner and collaborators suggested that RDR2 and PolIV may be responsible for intracellular silencing signalling rather than TGS in recovered leaves (Kørner et al. 2018).

**Fig. 1:**
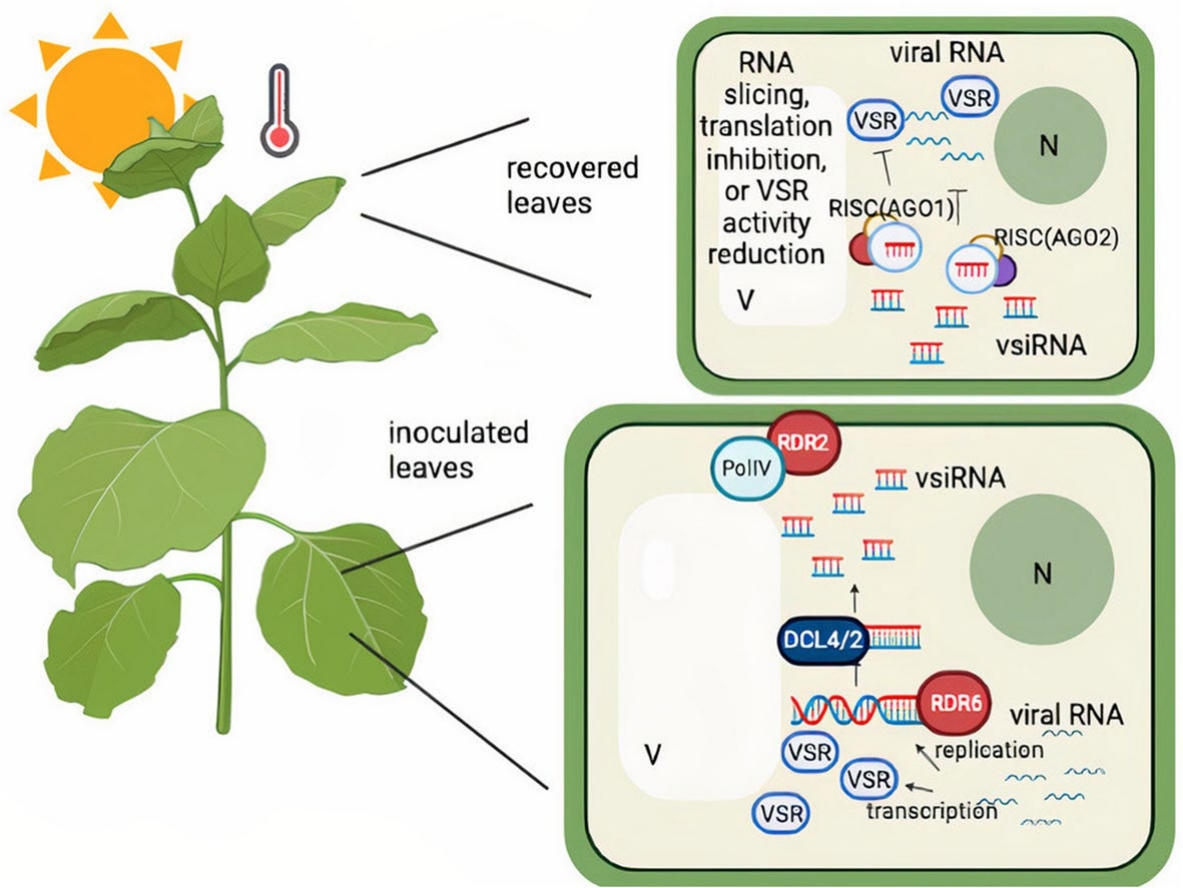
Model depicting the concept of symptom recovery at elevated temperature. Higher temperatures facilitate viral RNA replication at early stages of infection in inoculated leaves. An early onset of RNAi is triggered by the higher accumulation of viral RNAs, leading to an increased accumulation of viral siRNAs (vsiRNAs). Symptom recovery occurs when secondary siRNAs travel to non-inoculated leaves to trigger RNA slicing, translational inhibition or lower the activity of viral RNA silencing suppressors (VSRs). Components, such as RNA- dependent RNA polymerase 6 (RDR6), RDR2, DICER-LIKE 2 (DCL2), DCL4, AGRONAUTE 1 (AGO1), AGO2, and PolIV are involved in this mechanism. V = vacuole; N = nucleus

In contrast to what was discussed above, the components and steps in RNAi pathways which are involved in symptom recovery induced by elevated temperatures were similar to but not the same as those involved in recovery in plant grown at their vegetative temperature optimum. In tomato ringspot virus (ToRSV)-infected *N. benthamiana*, recovery at elevated temperature was concomitant with a faster accumulation of viral RNA2 and vsiRNAs in the early stages of infection. After initiation of symptom recovery, a similar level of RNA2 was observed at both 27 °C and 21 °C. Despite a higher or similar level of RNA2 in ToRSV-infected *N. benthamiana* at 27 °C compared to 21 °C, a lower level of RNA2-encoded proteins, e.g., the CP, was observed by in vivo labelling experiments. Furthermore, silencing of *Ago1* gene was shown to prevent symptom recovery and could enhance translation rate of CP at 27 °C (Ghoshal and Sanfacon 2014). Since CP is a weak VSR that can suppress AGO1-directed translational inhibition, a lower level of CP may reduce suppression of translational inhibition, which may lead to symptom recovery in ToRSV-infected *N. benthamiana* at 27 °C (Ghoshal and Sanfacon 2014). Similar to recovery shown in TRV-infected *N. tabacum*, recovery induced by elevated temperatures was concomitant with RNA or DNA reduction in some pathosystems, such as CP RNA of CMV-infected *N. tabacum*, viral RNA of capsicum chlorosis virus-infected capsicum and viral DNA of East African cassava mosaic-infected cassava (Chellappan et al. 2005; Tsai et al. 2022; Zhao et al. 2016). Moreover, vsiRNAs accumulated higher in these pathosystems at elevated temperature. According to the mechanisms or components involved in natural- and heat-induced recovery and heat-enhanced RNAi (Ghoshal and Sanfacon 2014; Ghoshal and Sanfacon 2015; Kørner et al. 2018; Qu et al. 2005; Zhang et al. 2012), an expanded model has been proposed to describe this temperature-dependent symptom recovery phenomenon at elevated temperatures (Paudel and Sanfacon 2018). First, elevated temperatures facilitate viral RNA replication (Ghoshal and Sanfacon 2014; Zhang et al. 2012), which may trigger the onset of antiviral RNAi at early stages of infection by providing an increase in the dsRNA trigger. Subsequently, vsiRNAs would accumulate to higher levels at elevated temperatures and in turn may move to systemically infected leaves to trigger viral RNA reduction, to block viral protein translation, or to block VSR activity (Ghoshal and Sanfacon 2014; Ghoshal and Sanfacon 2015; Kørner et al. 2018). Together these events would lead to symptom recovery in newly developing leaves (Fig. 1).

Unlike the previous examples, where elevated temperatures enhance plant antiviral RNAi, an increase in temperature may conversely weaken plant defence responses to facilitate virus infection in some pathosystems (Table 1) (Anfoka et al. 2016; Makarova et al. 2018; Obrepalska-Steplowska et al. 2015; Prasch and Sonnewald 2013). An inverse correlation between salicylic acid (SA)-mediated signalling and high temperature enhanced virus accumulation in thermo-sensitive/PVY-susceptible potato interaction (Makarova et al. 2018). SA is a vital phytohormone that mediates defence responses to viruses in both susceptible and resistant plants (Love et al. 2005; Vlot et al. 2009). SA can modulate multiple defence responses, including the up-regulation of pathogenesis-related (PR) genes or the enhancement of RNA silencing (Alamillo et al. 2006; Qi et al. 2018). The temperature-mediated enhancement of PVY infection in potato at 28 °C was associated with reduced induction levels of SA-induced PR-1b and PR-2, which are highly expressed in thermo-tolerant/PVY-resistant potatoes (Makarova et al. 2018). Similarly, high temperature of 32 °C, which compromised virus-induced up-regulation of PR genes, enhanced the replication of TuMV in *A. thaliana* (Prasch and Sonnewald 2013). Interestingly, PR proteins were less important than heat shock factors (HSFs) and heat shock proteins (HSPs) in tomato plants’ defence against combined stresses of tomato yellow leaf curl virus (TYLCV) and heat (Anfoka et al. 2016). In TYLCV-susceptible tomato plants, the levels of HSFs and HSPs were reduced during TYLCV infection, which may lead to a decreased tolerance of tomato plants to high temperature. This reduced stress response may in turn favour virus replication and enhance susceptibility of tomato plants to TYLCV (Anfoka et al. 2016). Overall, there is no one-size-fits-all viral pathosystem that can be used as a general model. Nevertheless, by analyzing the effects of elevated temperatures on different pathosystems, we can increase our knowledge and develop more resilient strategies for plant protection during multiple stresses, which will be discussed in the next section.

### Effect of elevated temperatures on the interactions between virus-resistant plants and viruses

In nature, sophisticated mechanisms are involved in plants’ survival when they are under virus attack. The major defense mechanisms of plants against viruses include ETI- mediated resistance and antiviral RNA silencing (Akhter et al. 2021; Baruah et al. 2020; Gallois et al. 2018). Unlike ETI that has long been viewed as an essential antiviral mechanism, pathogen-associated molecular pattern (PAMP) triggered immunity (PTI) was found to be elicited by virus infection only recently (Leonetti et al. 2021; Moon and Park 2016; Niehl et al. 2016). Besides those mechanisms, recessive resistance is a type of resistance where host plants are lacking essential factors for viruses to complete their life cycle (Akhter et al. 2021). In addition, Quantitative Trait Loci (QTL)-mediated quantitative resistance has been shown to be critical for the durability of plant resistance through modulating the efficiency of major-effect resistance genes (Gallois et al. 2018). Although most of the known resistance QTLs against viruses are yet to be cloned (Pilet-Nayel et al. 2017), examples suggest that QTLs can correspond to two categories of major-effect resistance traits mentioned above, resistance (R) gene-mediated resistance and recessive resistance (Caranta et al. 1997; Gallois et al. 2018; Ruffel et al. 2002; Ruffel et al. 2006).

Canonical PTI, which was initially recognized as the first line of defense against non-viral pathogens, is triggered by detection of conserved PAMP or damage associated molecular patterns through plant membrane-associated pattern recognition receptors (PRRs) (Monaghan and Zipfel 2012). Recently, the involvement of PTI in plant antiviral defense response has been proposed based on several lines of evidence (Korner et al. 2013; Li et al. 2019; Nicaise and Candresse 2017; Niehl et al. 2016; Zvereva et al. 2016). Firstly, PTI triggered by non-viral PAMPs could confer plants resistance to viruses (Korner et al. 2013; Li et al. 2019). Similar to non-viral pathogens, some viral encoded proteins, i.e., P6 of cauliflower mosaic virus and CP of plum pox virus, have been identified to serve as effectors to suppress PTI (Nicaise and Candresse 2017; Zvereva et al. 2016). Lastly, dsRNA-like molecules of viral origin were found to trigger PTI responses instead of RNA silencing through involvement of known PTI components (Niehl et al. 2016). However, the question of recognition of intracellular dsRNA by surface-localized PRRs still remains to be solved.

**Fig. 2:**
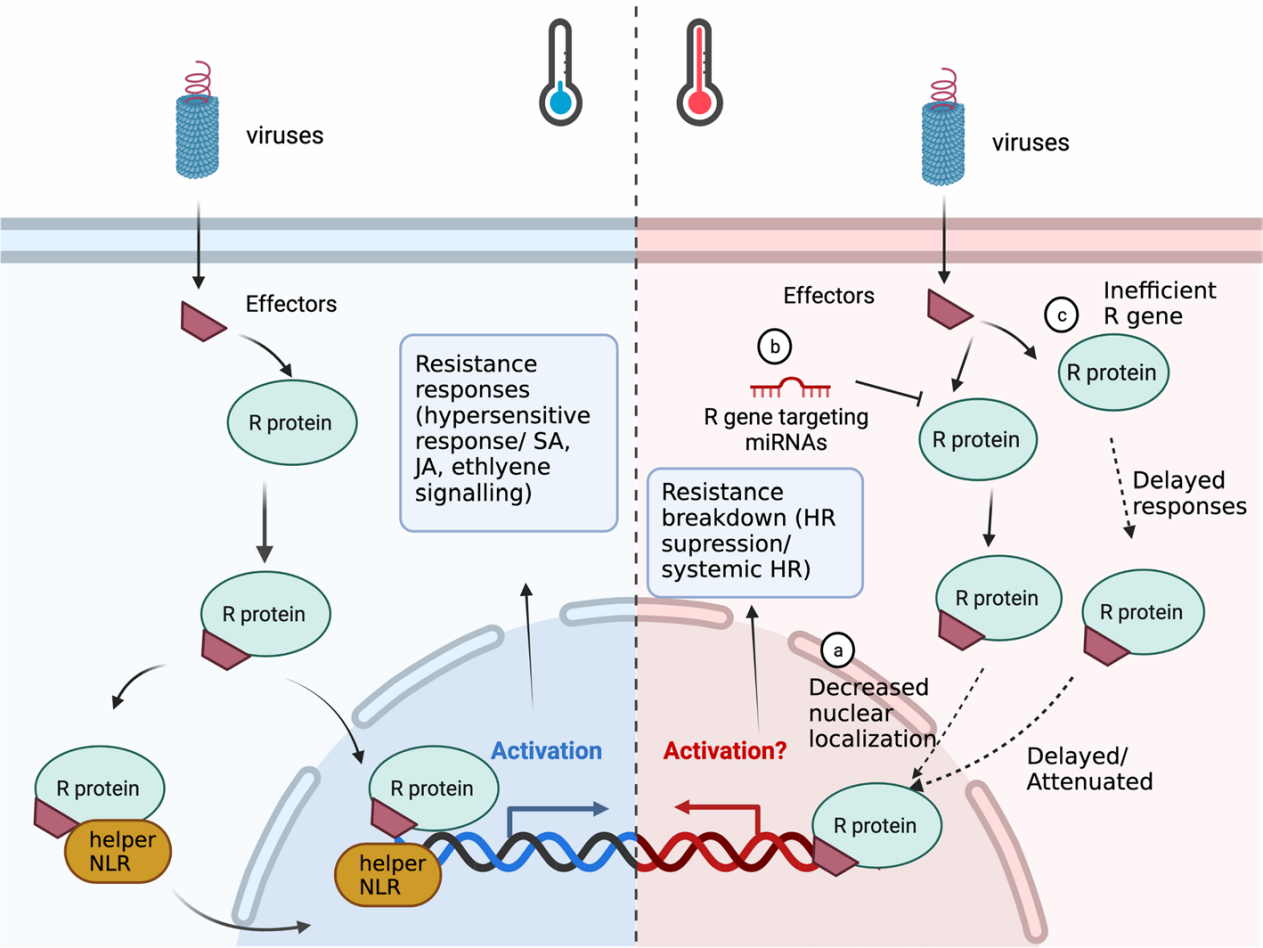
Schematic representation of plant antiviral resistance pathways (left) and resistance-breaking pathways (right). Activation of plant resistance responses is triggered by the recognition of viral effectors and plant resistance (R) proteins. For the resistance response, activated R proteins, belonging to nucleotide-binding leucine-rich repeat (NLR) type, may translocate to the nucleus or pair with helper NLRs to trigger downstream responses, such as hypersensitive response (HR), salicylic acid (SA), jasmonic acid (JA), and ethylene signalling (left). Under elevated temperature condition, a suppression or delay of resistance responses may be caused by **a** decreased nuclear localization of R proteins, **b** miRNA-mediated R gene degradation, or **c** insufficient R proteins, leading to resistance-breaking phenotypes, including HR suppression and systemic HR

To date, the majority of characterized R genes belong to the nucleotide-binding and leucine-rich repeat (NLR) type. These NLR proteins contain a nucleotide-binding (NB) domain, a C-terminal leucine-rich repeat (LRR) domain and a N-terminal domain (Cesari 2018). During virus infection, NLR proteins recognize pathogen effectors, which in turn trigger a robust ETI-mediated plant defense response (Gouveia et al. 2017). Since PTI was recently recognized as an antiviral mechanism, the ETI- and PTI- associated zigzag model that was first proposed as a concept for the arms race between host plants and non-viral pathogens (Jones and Dangl 2006) has also been adapted to fit viral resistance (Calil and Fontes 2016; Gouveia et al. 2017; Leonetti et al. 2021). Briefly, the proposed mechanism of this adapted model starts with the recognition of viral dsRNA, which in turn induces not only RNA silencing but also PTI against viral pathogens (Leonetti et al. 2021; Moon and Park 2016; Niehl et al. 2016). Subsequently, viral effectors are expressed by viral pathogens to concurrently counteract PTI. In the next phase, plants activate ETI to overcome effector-triggered susceptibility through recognition of the viral effectors or VSR proteins by NLR proteins (Leonetti et al. 2021; Moon and Park 2016; Niehl et al. 2016). Subsequently, calcium, SA and ROS rapidly accumulate to trigger two typical manifestations of ETI, which are the hypersensitive response (HR) and systemic acquired resistance (SAR), to confer plant resistance to viruses (Fig. 2) (de Ronde et al. 2014). Recessive resistance is achieved by recessive alleles of host genes that are essential for plant viral infection. Recessive resistance genes have been comprehensively reviewed (Akhter et al. 2021), and many have been shown to encode eukaryotic translation initiation factors (eIF) of the 4E or 4G type (elF4E/elF4G), and their isoforms (eIF (iso)4Es) (Akhter et al. 2021; Hashimoto et al. 2016). Examples of eIF4E-mediated recessive resistance are illustrated for potyviruses in mutants of *A. thaliana* (Lellis et al. 2002), *C. annuum*, *Lactuca sativa* and *S. habrochaites* (Nicaise et al. 2003; Ruffel et al. 2002; Ruffel et al. 2005). eIF4E-mediated recessive resistance was also identified against other RNA viruses, including CMV in *A. thaliana*, TCV in *A. thaliana*, melon necrotic spot virus in muskmelon, and rice yellow mottle virus in rice (Hashimoto et al. 2016).

Environmental variables, including temperature, need to be considered as factors affecting viral pathogenicity and degrees of plant resistance when plants carry major-effect resistance genes in different genetic backgrounds (Gallois et al. 2018). Very recently, the effects of elevated temperatures on plant resistance to a range of pathogens have been comprehensively reviewed (Desaint et al. 2021). Among 45 studies or reviews considered, either positive, negative or neutral effects on plant resistance were identified in 36 viral and non-viral pathosystems (Desaint et al. 2021). In the cited review, temperature effects on viruses in 11 pathosystems were listed by the authors. Importantly, 9 out of those 11 cases showed negative effects, and only the remaining 2 cases showed neutral effects on plant resistance to viruses at elevated temperatures (Desaint et al. 2021). This suggests that resistance is more likely broken down by viruses at elevated temperatures. Notably, breakdown of plant virus resistance has not only been seen at elevated temperature conditions. The emergence of resistance-breaking (RB) variants of viruses that can overcome plant major-effect resistance has been considered to be the main cause (Keller et al. 1998; Masuta et al. 1999; Nicolas et al. 1997). A group of RB variants that overcome recessive resistance was identified in several potyviruses that have natural variations in the genome-linked protein (VPg) (Gallois et al. 2018). Since the interaction of VPg and eIF4E is the key for potyvirus infection (Nicaise et al. 2003; Ruffel et al. 2002; Sanfaçon 2015; Wang and Krishnaswamy 2012), mutations in VPg can allow viruses to restore compatible interactions with host susceptibility factors and overcome the recessive resistance (Gallois et al. 2018). Besides breakdown of recessive resistance, another group of naturally occurring variants of viruses have been shown to break down R gene-mediated resistance (Gallois et al. 2018; Widyasari et al. 2020). This RB is generally attained through mutations in viral effectors, thereby avoiding plant R protein recognition (Gallois et al. 2018; Widyasari et al. 2020).

However, the underlying mechanisms involved in the breakdown of NLR-mediated resistance at elevated temperatures remain poorly understood in viral pathosystems (Szajko et al. 2019; Wang et al. 2009). So far, the most widely studied temperature-sensitive NLR-mediated resistance is the Suppressor of NPR1–1 Constitutive 1 (SNC1)-mediated resistance in a bacterial pathosystem (Venkatesh and Kang 2019). SNC1 confers resistance to the bacterium *Pseudomonas syringae* at 22 °C in *A. thaliana*, however the resistance is reversibly inactivated at 28 °C or above (Yang and Hua 2004). Multiple mechanisms are reportedly involved in this temperature sensitivity. One involves the increased accumulation of abscisic acid (ABA) at elevated temperature, which contributes to a decreased nuclear localization of SNC1 protein (Mang et al. 2012; Venkatesh and Kang 2019). This decreased accumulation prevents plants from mounting an effective HR response since the translocation of SNC1 into the nucleus is essential to orchestrate plant immune signalling (Mang et al. 2012; Venkatesh and Kang 2019; Wang et al. 2009). Another mechanism that causes temperature sensitivity is mediated by the accumulation of Phytochrome Interacting Factor 4 (PIF4), which may repress SNC1-mediated resistance at elevated temperatures (Gangappa et al. 2017).

In viral pathosystems, possible mechanisms involved in temperature sensitivity were identified in TMV-infected tobacco and PVY-infected potato plants (Table 2) (Szajko et al. 2019; Wang et al. 2009). Tobacco plants mount an effective HR when host R protein, N, accumulates in the nucleus after recognition of TMV p50 at 22 °C, while plants are unable to induce an HR at 28 °C. This temperature-sensitive resistance may be associated with the decreased N protein nuclear localization (Wang et al. 2009; Zhu et al. 2010) (Fig. 2, right panel (a)). On the other hand, plant microRNAs (miRNAs), a group of plant endogenous small RNAs that specifically regulate gene expression by target cleavage or translational inhibition (Borges and Martienssen 2015), appear to be linked to temperature sensitivity of Ny-DG-mediated resistance to PVY in potato plants. At 28 °C, Ny-DG-mediated resistance was compromised and an enhanced miRNA-mediated down-regulation of a specific NLR transcript was observed in PVY- inoculated potato leaves (Szajko et al. 2019) (Fig. 2, right panel (b)).

It appears that in general an absence of HR is associated with resistance breakdown (Desaint et al. 2021). However, several studies have shown that HR is neither required nor sufficient for plant resistance to viruses or other plant pathogens (Bendahmane et al. 1999; Bulgarelli et al. 2010; Menna et al. 2015; Takahashi et al. 2012; Yu et al. 1998). For example, the *dnd1* mutant line of *A. thaliana* was shown to retain resistance to avirulent *P. syringae*, even though the plant was unable to mount HR-based cell death (Takahashi et al. 2012; Yu et al. 1998). Another example was revealed in the *A. thaliana – P. syringae* pathosystem. The NLR resistance proteins ZAR1 and RPS2 recognize the type three effectors AvrRpt2 and HopZ1a secreted by *P. syringae*, which triggers ETI-mediated resistance and HR at ambient temperature. At an elevated temperature of 30 °C, HR was suppressed in most plants, while resistance remained unaffected (Menna et al. 2015). Interestingly, this disconnect between HR and disease resistance has also been seen in temperature-sensitive resistance to viruses (Chung et al. 2018; de Ronde et al. 2019; Moury et al. 1998). The *Tsw* gene-mediated resistance to TSWV in capsicum is compromised at 32 °C, resulting in virus movement to uninoculated leaves. Local HR was still triggered but was unable to restrict virus movement to inoculated leaves, which led to either systemic HR (SHR) or the typical TSWV systemic symptoms at 32 °C (Chung et al. 2018; de Ronde et al. 2019; Moury et al. 1998). While the mechanisms that underpin SHR caused by temperature-sensitivity remain largely unknown, a recent study provides some evidence that inefficient induction of resistance contributes to a switch from HR to SHR (Abebe et al. 2021) (Fig. 2, right panel (c)). This decrease in resistance induction results in unrestricted virus systemic movement, which upon virus arrival triggers HR in systemically infected leaves.

Besides negative effects, both neutral and positive effects of elevated temperatures on plant resistance to viruses have been seen in several cases (Desaint et al. 2021). Heat-stable resistance conferred by R genes, *L*^*1a*^ and *wsm3*, were identified in the tobacco mild green mosaic virus-capsicum and wheat streak mosaic virus-wheat pathosystems, respectively (Friebe et al. 2009; Sawada et al. 2004). In contrast to these two examples, R gene-mediated resistance of capsicum plants to TMV pathotype-P_0_ (TMV- P_0_) was suppressed at 34 °C. Interestingly, instead of completely compromising resistance at this elevated temperature, plants retained resistance to TMV through enhanced antiviral RNA silencing and exhibited no HR on inoculated leaves (Kim et al. 2021). At elevated temperatures, some temperature-dependent vsiRNAs were triggered in TMV-infected capsicum plants, which in turn conferred resistance to TMV- P_0_ (Kim et al. 2021). As highlighted in the above examples, the effects of elevated temperatures on plant resistance to viruses and the mechanisms involved in such temperature effects vary in different combinations of host plants and viruses. A more comprehensive understanding is therefore required and a need to broaden future studies to investigate so far unexplored research questions.

## Promising approaches for managing plant virus diseases under elevated temperature stress

Current approaches to manage plant virus diseases include phytosanitary measures, cultural and chemical control, natural host resistance, and biological control (Jones and Naidu 2019). Viral epidemics may be successfully managed by an appropriate combination of those individual measures (Jones and Naidu 2019). However, the efficacy of plant virus control measures has become more unpredictable due to increasing temperatures (Fu et al. 2020; Jones and Naidu 2019). For example, at elevated temperatures, some viruses can overcome the host resistance that was incorporated by antiviral breeding strategies (Chung et al. 2018; de Ronde et al. 2019; Liu et al. 2011; Moury et al. 1998; Sawada et al. 2004; Szajko et al. 2019). Since resistance breeding is known to be laborious and time-consuming, resistance breakdown caused by elevated temperatures brings additional challenges to this virus disease control approach. RNAi-based approaches, on the other hand, could complement or supplement conventional antiviral approaches since an enhancement of antiviral RNAi was seen in various pathosystems at elevated temperatures (Chellappan et al. 2005; Ghoshal and Sanfacon 2014; Kim et al. 2021; Qu et al. 2005; Siddiqui et al. 2008; Szittya et al. 2003; Velázquez et al. 2010; Zhang et al. 2012; Zhao et al. 2016). RNAi-based virus control approaches have been largely achieved through genetic engineering, which generates GM plants that are poorly accepted by the public in many countries (Dalakouras et al. 2020; Khalid et al. 2017; Rank and Koch 2021). On the other hand, exogenous applications of RNAi-based approaches have also been successfully applied to plants to trigger RNAi. Among those exogenous applications, “spray induced gene silencing” is a recent breakthrough to protect plants from viruses without the use of GM technology (Rank and Koch 2021; Taliansky et al. 2021). To broaden the strategies that can be exogenously applied to plants for controlling viruses, potential RNA-based techniques that were shown to work for crop protection through stable or transient expression will be discussed below.

Recently, another promising strategy based on nanoparticle technology has been developed to manage plant diseases with the goal of reducing degradation, leaching, run-off, or volatilization of active ingredients (Fu et al. 2020). This strategy involves nanocarriers for efficient delivery of pesticides and genetic materials into plants (Fu et al. 2020). The combination of RNAi-based techniques and nanoparticles has been shown to be a promising advance for viral disease management in the near future (Fletcher et al. 2020). This would pave the way for managing plant virus diseases by field application of sprayable RNA to combat elevated temperature stress.

### RNA-mediated regulation confers plant resistance to viruses

RNA-mediated techniques have been reported to confer plant resistance to various viruses (Taliansky et al. 2021; Teotia et al. 2016). Among these techniques, RNAi-based approaches have been particularly effective at controlling plant virus diseases (Koch and Wassenegger 2021; Rank and Koch 2021). RNAi is achieved or initiated by the delivery of an RNA silencing construct, which harbours sequence-specific RNA silencing inducers, such as sense RNA, antisense RNA, hairpin RNA (hpRNA), artificial miRNA (amiRNA), or artificial trans-acting siRNA (atasiRNA) (Khalid et al. 2017). To trigger RNAi specifically, nucleotide sequences targeting an untranslated region or a coding region (e.g., CP, nucleocapsid protein, viral replicase, or VSR) of the viral genome are included in the RNA silencing constructs (Khalid et al. 2017). Then, sRNAs, having complementary sequences to viral RNAs, are transcribed *in planta* to target viral RNAs and trigger RNA slicing. Examples of successful antiviral resistance induced by RNAi-based approaches have been comprehensively reviewed by several authors (Cillo and Palukaitis 2014; Khalid et al. 2017; Zhao et al. 2020). For instance, CMV-resistant tomato and TSWV-resistant tomato were generated by transgenic expression of targeted amiRNA and atasiRNA, respectively (Zhang et al. 2011). Furthermore, transgenic PVX-resistant potato, and CMV-, and TYLCV- resistant tomato were generated by overexpressing siRNAs targeting these viruses (Antignus et al. 2004; Doreste et al. 2002; Nunome et al. 2002). So far, RNAi-based approaches in crop protection have largely been achieved through genetically-engineering based strategies (Khalid et al. 2017). Moreover, some of those GM plants, such as papaya ringspot virus-resistant papaya, plum pox virus (PPV)-resistant plum, CMV- and ZYMV-resistant squash, PVY- and PLRV- resistant potato, CMV-resistant capsicum, CMV-resistant tomato, and bean golden mosaic virus-resistant common bean have been commercially released (Aragão et al. 2013; Khalid et al. 2017). Interestingly, antiviral resistance triggered by RNAi can be achieved not only by targeting the viral genome, but also by targeting virus susceptibility genes in plants (Taliansky et al. 2021). One example are plum plants that were engineered to express hpRNA targeting eIF4E or eIF (iso)4E. Since eIF4Es are host genes that are essential for potyvirus infection by interacting with VPg, silencing either of them partially protected plants from PPV infection (Wang et al. 2013b). Another example are transgenic coilin-silenced tobacco plants (Shaw et al. 2014). Coilin, the structural protein that is essential for Cajal bodies formation, was found to enhance the susceptibility of tobacco plants to PVY. As a result of coilin silencing, PVY titre was significantly decreased (Shaw et al. 2014).

Another RNA-mediated technique, termed target mimics (TM), has been used to attract or decoy sRNAs instead of amplifying them (Teotia et al. 2016). TM was discovered as an endogenous mechanism of sequestering miR399 in *A. thaliana* (Franco-Zorrilla et al. 2007). *INDUCED BY PHOSPHATE STARVATION 1* (*IPS1*) is a non-coding gene that is transcribed into non-coding RNA containing a sequence that is partially complementary to miR399; three nt mismatches are located in the expected miR399 cleavage site. When miR399 pairs with *IPS1* transcripts, the 3 nt mismatches in *IPS1* transcripts form a central bulge to block the activity of miR399. Therefore, miR399 is arrested by TM (*IPS1*) and fails to cleave its intended target *PHOSPHATE2* (Franco-Zorrilla et al. 2007; Teotia et al. 2016). Subsequently, an improved approach, named short tandem target mimics (STTM), was developed based on TM (Tang et al. 2012; Yan et al. 2012). STTM are weak RNA stem-loop structures, harbouring two sRNA target-mimicked sequences linked by a 48 nt or 88 nt spacer (Yan et al. 2012). Different from TM-mediated miRNA arrest, STTM block miRNA activity by inducing sRNA-degrading nucleases that contribute to 3′-truncated miRNAs and miRNA degradation (Yan et al. 2012). To date, the STTM approach has been applied to improve resistance to plant diseases by inhibiting or dampening miRNA activity in several crop plant species (Bao et al. 2018; Canto-Pastor et al. 2019; Jiang et al. 2018). For example, resistance to *Phytophthora infestans* and *P. syringae* was achieved in STTM482/STTM2118b - transgenic tomato plants (Canto-Pastor et al. 2019; Jiang et al. 2018). This may have been due to blocking of miR482/2118 and reduced silencing of their NLR targets, resulting in enhanced quantitative disease resistance (Canto-Pastor et al. 2019; Jiang et al. 2018). Furthermore, inhibition of miR482a by transiently overexpressed STTM was shown to compromise soybean mosaic virus infection in soybean (Bao et al. 2018).

Clustered regularly interspaced short palindromic repeat (CRISPR)-associated protein 9 (CRISPR-Cas9) is a dual-factor system that consists of single guide RNA (sgRNA) and Cas9 DNA endonuclease (Ebrahimi and Hashemi 2020). A complex formed by Cas9 nuclease and sgRNA binds to target DNA through recognition of a specific sequence by sgRNA. Subsequently, Cas9 nuclease creates a DNA double-strand break (DSB) at the target site, which is then repaired to yield either deletions or insertions (Ebrahimi and Hashemi 2020; Taliansky et al. 2021). The applications of this concept have been expanded since the discovery of additional sgRNA-guided nucleases (Koonin et al. 2017). Rather than causing DSBs, FnCas9 and Cas13 for example are directed by sgRNA to mediate ssRNA cleavage at the target RNA site (Koonin et al. 2017). Based on the different specificities of Cas nucleases, CRISPR-Cas9 and CRISPR-FnCas9/CRISPR-Cas13 systems have been applied to control DNA and RNA viruses, respectively (Ali et al. 2016; Aman et al. 2018; Kis et al. 2019; Liu et al. 2018; Zhan et al. 2019; Zhang et al. 2018). Resistance to DNA viruses, including TYLCV in *N. benthamiana* (Ali et al. 2016), wheat dwarf virus in barley (Kis et al. 2019), and CaMV in *A. thaliana* (Liu et al. 2018), was successfully conferred by transgenic expression of CRISPR-Cas9. Transgenic plants expressing CRISPR-FnCas9 or CRISPR-Cas13 were reported to be resistant to RNA viruses, including CMV and TMV in tobacco (Zhang et al. 2018), PVY in potato (Zhan et al. 2019), and TuMV in *N. benthamiana* (Aman et al. 2018). Similar to RNAi-based approaches, the CRISPR-Cas9 system was also successfully used to edit virus susceptibility genes, such as *coilin*, to enhance resistance to PVY in potato (Makhotenko et al. 2019) and *eIF4E2* to generate resistance to pepper veinal mottle virus in cherry tomato (Kuroiwa et al. 2022).

Although these RNA-based approaches have not yet been exploited to reduce temperature-sensitivity of virus resistance, they could be viewed as promising strategies for minimizing the risk of resistance-breakdown at elevated temperatures. Significantly, plants exhibit more effective antiviral RNA silencing at higher temperatures than at lower temperatures. Further, miRNA-mediated R gene regulations may negatively affect plant resistance to viruses. This suggests that RNAi-based approaches that target viral RNAs or STTMs that target R gene-targeting miRNAs may overcome the resistance-breakdown caused by elevated temperatures.

### Exogenous application of RNA-based approaches for plant protection

The RNA-mediated techniques mentioned above have been primarily delivered by stable transgenesis, Agrobacterium-mediated transient overexpression, or virus-induced gene silencing (Khalid et al. 2017; Koch and Kogel 2014; Rosa et al. 2018; Zotti and Smagghe 2015). However, external application of dsRNA was first shown to successfully trigger RNAi in tobacco and pepper plants by mechanical inoculation of leaves with dsRNA targeting pepper mild mottle virus (PMMoV), TEV and alfalfa mosaic virus (Tenllado and Diaz-Ruiz 2001). Since then, many reports have demonstrated that it is possible to externally apply pest−/pathogen-specific long dsRNA, hpRNA, or siRNA for RNAi-mediated control (Cagliari et al. 2019; Dubrovina and Kiselev 2019). Delivery of silencing RNAs generated by in vitro transcription or bacterial expression has been achieved by various methods, including foliar application, petiole absorption, trunk injection, and irrigation (Cagliari et al. 2019; Dubrovina and Kiselev 2019). Interestingly, the route of RNA translocation and the efficiency of RNAi inside plant cells can differ depending on the method of external RNA application (Dalakouras et al. 2020). High-pressure spraying of 685-bp dsRNA or 21-nt sRNAs with surfactant pre-treatment on *N. benthamiana* leaves delivered the RNA into plant cells and achieved both local and systemic RNAi (Sammons et al. 2011). On the other hand, RNAi was not triggered in plants by either sRNA or hpRNA applications through petiole absorption and trunk injection (Dalakouras et al. 2018). Instead, intact RNAs were retained in the xylem and mounted efficient RNAi inside insects that ingested xylem transported RNAs (Dalakouras et al. 2018).

For virus control, it is essential at some point to deliver RNAs into the plant cells. Once RNA has entered the cell, symplastic transport of these RNAs would lead to the onset of systemic RNAi (Dalakouras et al. 2020). To date, foliar applications of dsRNAs, hpRNAs, and siRNAs, triggering RNAi to protect plants from viruses through spray, infiltration, carborundum-dusted rubbing, and spreading using a pipette or brushes, have been well documented and reviewed in several studies (Dubrovina and Kiselev 2019; Konakalla et al. 2016; Tenllado and Diaz-Ruiz 2001; Tenllado et al. 2003; Yin et al. 2009). However, these treatments, except for spraying are only applicable at the laboratory scale but not at the field scale. Moreover, the short period of antiviral protection due to a rapid degradation of sprayable naked RNAs in the environment is a major concern for field applications. Recently, this limitation was addressed by binding the RNA to positively charged carrier nanoparticles (NPs) (Mitter et al. 2017). The mechanisms of uptake and translocation of NPs after foliar application are dictated by several factors including application methods, NP size and concentration, and the environment (Ali et al. 2021; Wang et al. 2013a). Generally, smaller NPs (between 10 and 50 nm) favour transport through the symplastic pathway into the vascular system, while larger NPs (between 50 and 200 nm) are translocated through the apoplastic pathway (Ali et al. 2021; Ruttkay-Nedecky et al. 2017). Moreover, the internalized NPs, absorbed into cells by endocytosis, or taken up by pore formation, carrier proteins, plasmodesmata or ion channels (Bao et al. 2017; Pérez-de-Luque 2017), are transported along the phloem sieve tubes in the vascular system (Ali et al. 2021). To date, Mg^2+^ and Al^3+^-based layered double hydroxides (LDHs), termed “BioClay”, have been successfully combined with dsRNAs and applied to protect tobacco plants from virus infection. Rather than being internalized, BioClay is broken down when exposed to atmospheric carbon dioxide and moisture. Therefore, this carrier served to protect dsRNAs from degradation on leaves rather than facilitate penetration of dsRNAs across the plasma membrane (Mitter et al. 2017). With the protection of dsRNA by BioClay, a continuous RNA supplement was provided to achieve long-lasting RNAi- mediated resistance against CMV and PMMoV for 20 days with a single topical application. Moreover, the application of dsRNA-BioClay complex protected tobacco both locally and systemically (Mitter et al. 2017; Worrall et al. 2019).

**Fig. 3:**
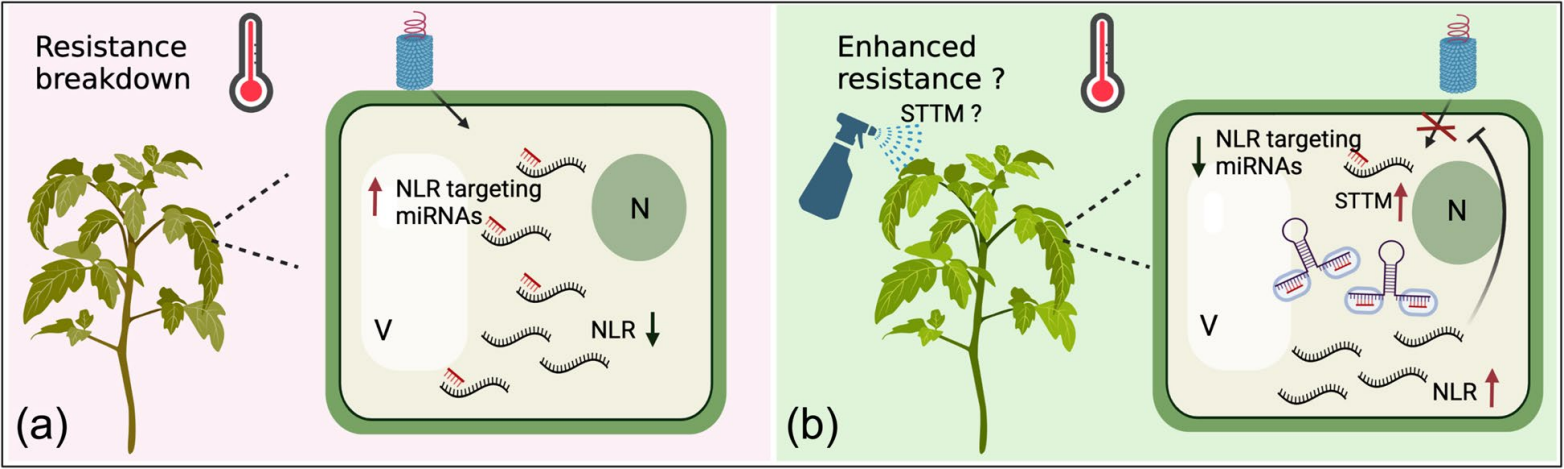
Schematic representation of exogenous application of short tandem target mimic (STTM) RNA molecules to degrade or sequester nucleotide-binding leucine-rich repeat (NLR) - targeting miRNAs. **a** At elevated temperatures, plant resistance to viruses may be suppressed by miRNA-mediated downregulation of NLRs. **b** STTM RNA molecules that target NLR-targeting miRNAs are delivered into the cytoplasm to reduce the abundance of miRNAs, which hence upregulates the expression of NLRs and enhances plant resistance. V = vacuole; N = nucleus.

**Fig. 4:**
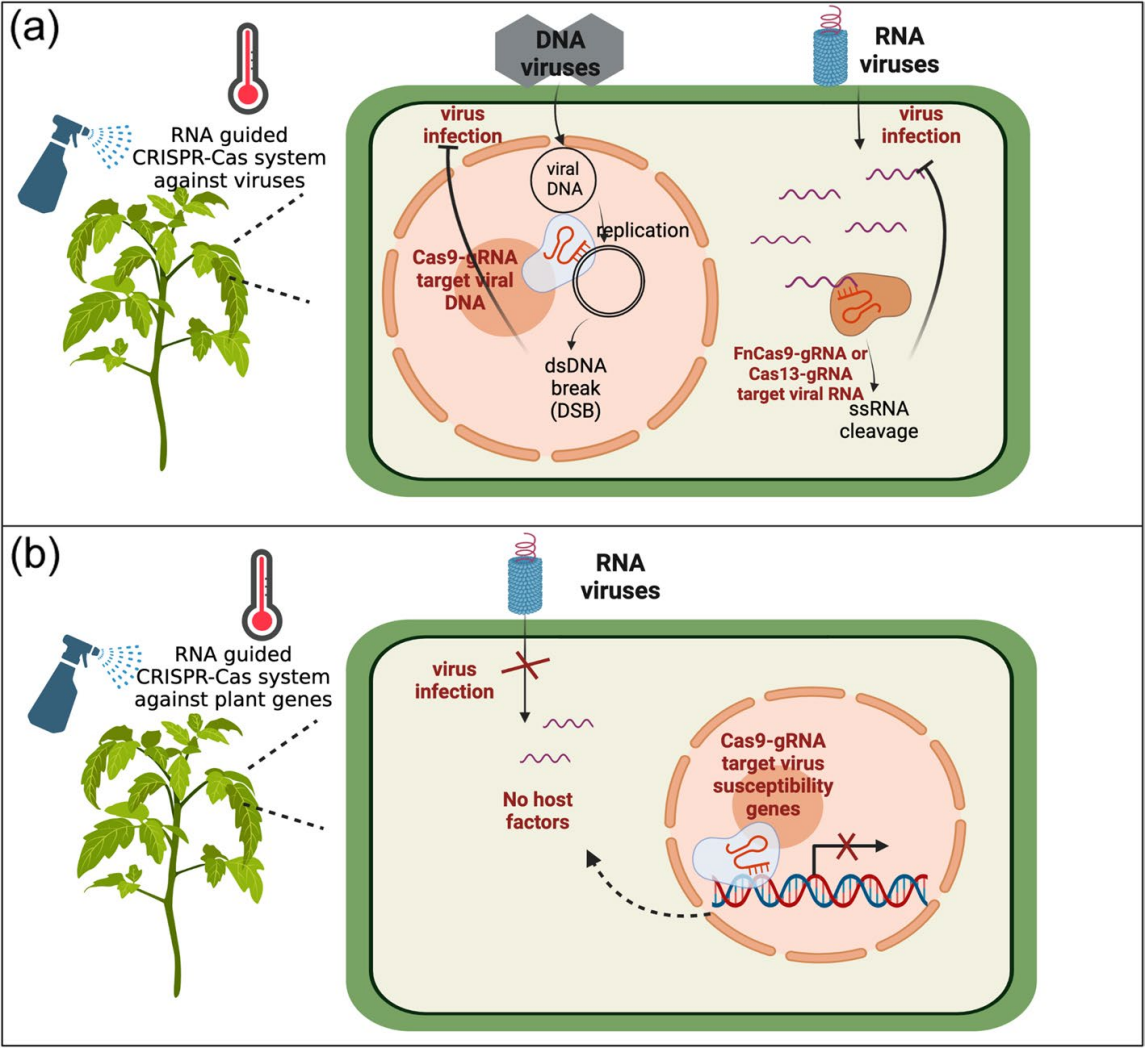
Schematic representation of proposed exogenous application of RNA guided CRISPR-Cas system to confer resistance to plant viruses. **a** CRISPR/Cas targets viral genomic DNA or RNA for destruction. **b** CRISPR/Cas targets virus susceptibility genes that are essential for viral infection of host

Other than delivering RNAs into plants, NPs have also been utilized for DNA delivery (Mujtaba et al. 2021). DNA-loaded mesoporous silica nanoparticles (MSN), single-walled carbon nanotubes (SWNT), and LDH may be internalized into intact plant cells without requiring strong mechanical aids (Bao et al. 2016; Kwak et al. 2019;  Chang et al. 2013). For example, MSN were used to deliver mCherry-encoding plasmid DNA (pDNA) into *A. thaliana* roots without external biolistic or other mechanical aids (Chang et al. 2013). This successful DNA delivery through nanoplatforms provides a potential new path for delivery of CRISPR-Cas9 pDNA. Delivery systems, including layer-by-layer self-assembling peptide coated nanofibers, poly (lactic-co-glycolic acid), and alginate nanoparticles, have shown promise as carriers for CRISPR-Cas9 pDNA (Alallam et al. 2020; Jo et al. 2020; Zhang et al. 2020). However, further investigations, such as applying these systems to plant cells and intact plants, are needed to determine their true potential for crop protection or gene editing. A possible application of STTM and CRIPR-Cas9 by spraying plants is proposed in Figs. 3 and 4. So far, the knowledge of delivering STTM and CRIPR-Cas9 through nanoplatforms is limited and the application of these tools is still in its infancy. This in turn indicates that the possibility of topical application of these two biomolecules at present is limited. However, given the latest advances in applications of RNA-mediated techniques and NPs in plant or animal systems (Damase et al. 2021; Mujtaba et al. 2021; Shin et al. 2018), nanoplatform-mediated RNA delivery appears to be a promising alternative to chemical pesticides in the future of sustainable agriculture.

## Conclusions and future perspectives

The ongoing increase in global temperatures undoubtedly will affect plant virus infections in various ways, which in turn will pose new threats to global food security. Elevated temperatures could influence virus epidemics at micro-climate and macro-climate levels. This includes changes in within-host virus accumulation and between-host virus transmission, which are impacted by changes in factors, such as vector performance (i.e., longevity, fecundity and population density), vector behaviors (i.e., dispersal, landing and feeding preference), virus movement and host plant traits. Notably, virus accumulation in individual plants can influence not only symptom expressions but also viral transmission, which emphasizes the importance of plant-virus interactions in virus epidemics. Given these considerations, gaining an overview of temperature effects on plant-virus interactions is highly critical for developing virus control strategies that are resilient to global warming.

The development of virus-resistant crop varieties has been a traditional and effective way to manage plant virus diseases in agriculture for decades. However, both conventional breeding strategies and strategies augmented with molecular techniques, including QTL mapping and marker-assisted selection, are laborious and time-consuming endeavours. Considering projected elevated temperature effects, it should be noted that plant breeding may be under more immense pressure than previously due to negative effects of higher temperatures on R gene-mediated resistance to viruses in many pathosystems. This anticipated breakdown of R gene-mediated resistance to viruses may decrease resistance durability, which in turn poses challenges for controlling plant virus diseases through breeding strategies at elevated temperatures. Owing to this concern, climate-resilient approaches, and robust platforms for capturing the most complete picture of genetic variations are needed to speed up the development of ‘temperature-insensitive’ virus-resistant crops or to lower the rate of resistance-breakdown at elevated temperatures.

Research that aims to increase plant resistance to viruses by applying nanocarrier-mediated modifications can potentially overcome the above challenges. Nanoplatforms for delivering biomolecules are also considered as promising substitutes to plant genetic engineering. As noted earlier, genetic engineering can be a relatively efficient approach to improve plant resistance or tolerance to biotic or abiotic stresses. However, generation of GM crops by genetic engineering is lacking broad public acceptance even though some GM crops have been commercialized for many years and shown to be safe. Considering this public acceptance issue and the challenges for conventional breeding, GM-free and less time-consuming NP delivery systems, therefore, attract much scientific and commercial attention. So far, sprayable RNA biopesticides that trigger RNAi are the most well-studied cargos delivered by NPs into plants for controlling plant viruses. Since antiviral RNAi has been shown to be enhanced by elevated temperatures in either virus-susceptible or virus-resistant plants, exogenous application of NP-conjugated RNA for SIGS induction appears to be a promising strategy for plant virus management in global warming scenarios. Interestingly, the choice of cargo for conjugated NPs may not be limited to siRNAs or dsRNAs, but may be expanded to miRNAs, CRIPR/Cas9 plasmids, or ssRNAs, such as STTM, making nanocarriers a versatile platform for virus control during climate change.

Due to the reality of inevitable changes of our climate, an increased effort to understand temperature effects on virus epidemiology and molecular plant-virus-vector interactions is needed. Considering the growing and comprehensive knowledge of molecular plant-virus-vector interactions, we believe a combination of RNA-based virus resistance technologies and NPs will provide promising strategies for virus control at elevated temperatures. Based on RNA-based analyses or capturing the landscape of genetic diversity, this strategy can be applied to trigger antiviral RNAi, regulate the expression of endogenous genes, or perform gene editing without the need to generate GM crops. Future studies should concentrate on understanding the efficiency of NP-mediated RNA-based technology at both lab and field scales, evaluating off-target risks, and optimising application time, to allow this strategy a sustainable success at agricultural field scale in the foreseeable future.

## Data Availability

Not applicable.

## Abbreviations

GM: Genetically modified
IPCC: Intergovernmental Panel on Climate Change
CP: Coat protein
MP: Movement proteins
vRNP: Viral ribonucleoprotein
ER: Endoplasmic reticulum
VRC: Virus replication complex
SEL: Size exclusion limit
ROS: Reactive oxygen species
ETI: Effector-triggered immunity
R gene: Resistance gene
RI: Resistance-inducing
TempRB: Temperature-dependent resistance-breaking
AbsRB: Absolute resistance-breaking
RNAi: RNA interference
DCL: Dicer-like proteins
siRNA: Small interfering RNA
vsiRNAs: Virus-derived siRNAs
AGO: Argonaute
RISC: RNA-induced silencing complex
PTGS: Post transcriptional gene silencing
TGS: Transcriptional gene silencing
VSR: Viral silencing suppressor
RDR: RNA-dependent RNA polymerase
HEN1: Hua Enhancer 1
GFP: Green fluorescent protein
SGS3: Suppressor of Gene silencing 3
NRPD: Nuclear RNA polymerase D
SA: Salicylic acid
PR: Pathogenesis-related
HSP: Heat shock protein
HSF: Heat shock factor
PAMP: Pathogen-associated molecular pattern
PTI: PAMP triggered immunity
QTL: Quantitative Trait Loci
PRR: Plant membrane-associated pattern recognition receptor
NLR: Nucleotide-binding and leucine-rich repeat
NB: Nucleotide-binding
LRR: Leucine-rich repeat
HR: Hypersensitive response
SAR: Systemic acquired resistance
eIF: Eukaryotic translation initiation factor
RB: Resistance-breaking
SNC1: Suppressor of NPR1–1 Constitutive 1
ABA: Abscisic acid
SHR: Systemic HR
hpRNA: Hairpin RNA
amiRNA: Artificial miRNA
atasiRNA: Artificial trans-acting siRNA
TM: Target mimics
IPS1: Induced by Phosphate Starvation 1
STTM: Short tandem target mimics
CRISPR: Clustered regularly interspaced short palindromic repeat
sgRNA: Single guide RNA
DSB: DNA double-strand break
NPs: Nanoparticles
LDH: Layered double hydroxide
MSN: Mesoporous silica nanoparticles
SWNT: Single-walled carbon nanotubes
pDNA: Plasmid DNA
JA: Jasmonic acid
Dpi: Days post inoculation
Wpi: Weeks post inoculation
NT: Normal temperature
LT: Lower temperature
HT: Higher temperature
Temp: Temperature
n.d.: Not determined
TSWV: Tomato spotted wilt virus
TMV: Tobacco mosaic virus
PVY: Potato virus Y
TuMV: Turnip mosaic virus
PMMoV: Pepper mild mottle virus
PPV: Plum pox virus
TYLCV: Tomato yellow leaf curl virus
ToRSV: Tomato ringspot virus
TRV: Tobacco rattle virus
TBSV: Tomato bushy stunt virus
ORMV: Oil-seed rape mosaic virus
CymRSV: Cymbidium ringspot virus
TEV: Tobacco etch virus
TRSV: Tobacco ringspot virus
ChiLCV: Chili leaf curl virus
PVX: Potato virus X
TriMV: Triticum mosaic virus
WSMV: Wheat streak mosaic virus
TMGMV: Tobacco mild green mosaic virus
PaMMV: Paprika mild mottle virus
TCV: Turnip crinkle virus
CPsV: Citrus psorosis virus
ACMV: African cassava mosaic virus
SLCMV: Sri Lankan cassava mosaic virus
EACMCV: East African cassava mosaic Cameroon virus
EACMV: East African cassava mosaic virus
GBNV: Groundnut bud necrosis virus
INSV: Impatiens necrotic spot virus
CaCV: Capsicum chlorosis virus
